# Algorithm for correcting optimization convergence errors in Eclipse

**DOI:** 10.1120/jacmp.v10i4.3061

**Published:** 2009-10-14

**Authors:** Albert S. Zacarias, Michael D. Mills

**Affiliations:** ^1^ Department of Radiation Oncology University of Louisville School of Medicine Louisville KY 40202 USA

**Keywords:** optimization, AAA, OCE, iterative correction

## Abstract

IMRT plans generated in Eclipse use a fast algorithm to evaluate dose for optimization and a more accurate algorithm for a final dose calculation, the Analytical Anisotropic Algorithm. The use of a fast optimization algorithm introduces optimization convergence errors into an IMRT plan. Eclipse has a feature where optimization may be performed on top of an existing base plan. This feature allows for the possibility of arriving at a recursive solution to optimization that relies on the accuracy of the final dose calculation algorithm and not the optimizer algorithm. When an IMRT plan is used as a base plan for a second optimization, the second optimization can compensate for heterogeneity and modulator errors in the original base plan. Plans with the same field arrangement as the initial base plan may be added together by adding the initial plan optimal fluence to the dose correcting plan optimal fluence. A simple procedure to correct for optimization errors is presented that may be implemented in the Eclipse treatment planning system, along with an Excel spreadsheet to add optimized fluence maps together.

PACS number: 87.53.Bn, 87.56.By

## I. INTRODUCTION

IMRT plans generated in Eclipse use a Dose Volume Optimizer (DVO) algorithm to evaluate dose for optimization, and a more accurate algorithm – the Analytical Anisotropic Algorithm (AAA) – for a final Volume Dose (VD) calculation. Optimization in Eclipse with the DVO is subject to optimization convergence errors (OCEs).^(^ ^1^ ^–^ ^4^ ^)^ The error is primarily due to dose calculation for lateral scatter, dose calculation in the buildup region, and errors due to modeling modulator transmission. Dose calculation errors are present in plans which have planning target volumes (PTVs) near regions of electronic disequilibrium. This includes PTVs in most head & neck presentations, as well as many lung cases. OCEs are not always apparent because in most clinical head and neck presentations, the volume of electronic disequilibrium near the surface as a fraction of the total PTV volume is small. A dose‐volume histogram (DVH) presents dose representations for the total PTV. IMRT plans with a PTV in the lung, on the other hand, may demonstrate large differences between the optimized DVO‐based DVH and the final AAA‐based dose calculation DVH.

The need to provide fast optimization, as in Eclipse's DVO, relies on simplified dose calculation engines which are less accurate than the final dose calculation. This process results in a less than optimal plan due to optimizer limitations. The discrepancy is usually noted by comparing the optimizer PTV DVH curve, which typically meets the objectives of optimization, with the PTV DVH obtained on final dose calculation. Since the errors are generally small, the most efficient procedure is to perform a large number of iterations within the DVO, followed by a few periodic corrections (2–4 times) with the more accurate final dose calculation. This method is applied here for the Eclipse DVO and final volume dose (VD) grid calculated with AAA. The procedure should also work for the final dose calculated with a Monte Carlo algorithm in place of AAA.

## II. MATERIALS AND METHODS

OCEs originating from the DVO may be corrected by a simple recursive procedure,^(5)^ summarized in this technical note. The procedure takes advantage of the base plan feature in Eclipse that can optimize an IMRT plan on an existing 3D dose distribution from another plan. An IMRT plan (call it “Plan 0”) calculated with AAA properly accounts for heterogeneity corrections and MLC transport. If a copy of the plan is reoptimized with the original Plan 0 as a base plan (call this result “Plan DC0”), the benefit of the accuracy of AAA is obtained within the optimization. Typically, the new optimization should add as little dose as possible to the sum plan as the additional dose is calculated with the DVO and retains its error. The new plan is a sum of Plan 0 and Plan DC0. The plan sum can be generated within Eclipse using the “Plan Sum” feature. The number of fields will increase on each recursion. In order to keep the number of fields the same, optimal fluence maps from both plans may be added together to provide a plan with the same number of fields as the original. The procedure may be repeated by using Plan (i) as a new base plan until the desired level of accuracy is achieved. The final plan is normalized in the same manner as any other IMRT plan using the DVH curve for the PTV at a volume point. This recursive method is facile to implement this in the clinic with the Eclipse TPS without using any novel or custom optimization algorithms. The ability to add a base plan dose on an IMRT plan optimization is a unique feature of Eclipse (Varian Medical Systems, Inc, Palo Alto, CA), which makes possible an optimal solution based on the treatment planning systems final dose calculation algorithm.

### A. recursive procedure for optimization

The sum of the dose distributions from Plan (0) and Plan DC(0), with suitable normalization, will provide a better optimized plan than the original Plan (0). The recursion formula is given by:

(1) Plan (i+1)=Plan (i)+PlanDC (i)

where Plan (i) is used as a base plan, PlanDC (i) is the optimized dose correction plan, and Plan (i+1) is the new plan. In the next iteration, Plan (i+1) becomes the base for a new dose correction. The symbol “Plan” refers to both the dose calculation and the optimal fluence maps of each plan. If the fields are common in all the plans, the symbolic process in Eq. 1 may be implemented to simplify treatment delivery by adding fluence maps from the respective plans together. The intermediate plans are all unnormalized (plan normalization=100%). The final plan is normalized as any other IMRT plan, typically by adjusting the normalization factor to provide the Rx dose at the 95% volume point.

### B. Example method with a phantom

An elliptical water phantom with dimensions 14cm×18cm is used in this example calculation. The PTV is a 10×10cm patch, 1 cm deep along the surface. The IMRT plan uses seven lateral fields. A static field IMRT plan, Plan (0), is optimized with DVO 8.2.23 and the final dose grid is calculated with AAA 8.2.23 for a prescription dose of 100 cGy. An initial optimization plan, PlanDC (0), is performed with all limits scaled by the ratio of the maximum PTV dose / Rx dose using the base plan, Plan (0). After optimization of 50–100 iterations for PlanDC (0) and a final dose calculation using the AAA algorithm with calculation grid set to 0.2 cm or less, the dose fluence maps for the fields in PlanDC (0) were added to the dose fluence maps for the corresponding treatment fields in Plan (0). Therefore, a sum plan is generated by adding the optimal fluence of each field for the unnormalized plans Plan (0) and PlanDC (0). A spreadsheet is available at http://journals.sfu.ca/multimed/jacmp/pages/fles/Add_Fluence_JACMP_Evaluated.xls to add these fluence maps together. This process is outlined in Table 1, for easy reference. This compensation of dose may be repeated any number of times to reduce optimizer OCEs to a negligible level. The PTV dose limits in the optimizer are always chosen to be equal to or slightly greater than the maximum dose in the sum plan. In principle, the recursive method presented here should converge more quickly when each successive plan adds as little dose as possible to the sum plan.

**Table 1:** Procedure for generating one plan from two separate plans having the same MLC ports using the “Add Fluence. xlsm” spreadsheet program. The plan names are arbitrary but the program assumes fields are named consecutively Field 1, Field 2, and Field 3… in Plan and PlanDC.

*Step*	*Procedure*
1	Create folders, Plan, PlanDC and Plan(i)
2	Export DICOM Plan and dose correction plan PlanDC to appropriate folders. Include fluence as an option when using the export wizard.
3	Use dcm2Ascii.exe (provided by Varian, Palo Alto, CA) to extract the optimal fluence from the DICOM Plan files into text files by running the application and specifying the DICOM file input. The *.optimal fluence files are placed in the same folder as the DICOM file.
4	Specify in the spreadsheet “N” the patient name, number of fields and the drive letter where the folders Plan and PlanDC are located.
5	Run the spreadsheet macro by clicking “Add Fluence”. The results are placed in the folder Plan(i).
6	In Eclipse, import the optimal fluence in folder Plan(i) into a copy of the original base plan by right clicking on the field and selecting “import optimal fluence”.

For Eclipse planning, the IMRT plans should be generated with sufficient resolution in the PTV structure (≤0.2cm), smoothing reduced in the X direction to at least 10, and the jaws fixed for each dose correction in the optimizer. After optimization, the leaf levels in the MLC leaf motion sequencer should be set to 100 and the final dose calculation grid with AAA set to 0.2 cm. These setting are necessary to ensure that the optimizer can generate a plan with the requisite modulation to achieve an optimal plan within the capability of the AAA algorithm. For instance, increasing the MLC levels to 100 ensures that, when plans are added together, the intensity modulation of the small dose corrections is not lost to round‐off when using only 10 levels. After completion of the last AAA dose calculation (2–3 recursions are usually sufficient), the MLC levels should be reduced to a practical value; typically, 20 levels are sufficient to provide most of the benefit of this dose correction procedure.

### C.1 Clinical case 1: scalp vertex

A pediatric case with a neuroblastoma metastasized to the bony calvarium from the forehead to the vertex of the skull has a CTV which extends to the patient's surface. An IMRT plan with gantry angles every 20° from 270° to 90° was optimized and corrected according to the same procedure as the phantom study. A normal tissue volume comprising the brain minus the PTV volume with a 0.5 cm margin was used as an avoidance structure. The prescription was for 400 cGy at the 95% volume point in five fractions.

### C.2 Clinical case 2: lung

Stereotactic body radiotherapy of a lung tumor in the right medial lobe was treated with six fields using gantry angles from 55° to 255° with a prescription of 5000 cGy to the 95% volume point in five fractions. Plans were optimized and corrected using the same procedure as the Phantom study. The normal tissue structure for avoidance was constructed from the right lung minus a volume of the PTV with a 0.5 cm margin.

## III. RESULTS

### A. Phantom

**Figure 1:**
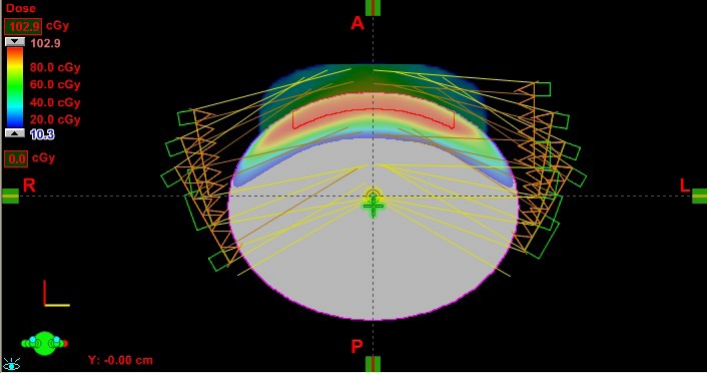
Water phantom generated in Eclipse showing PTV and 7‐field arrangement used in this study.

**Figure 2:**
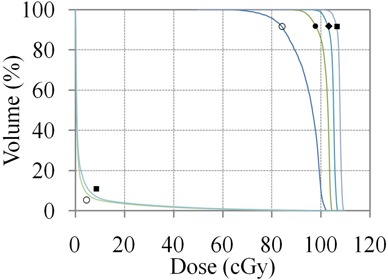
DVH curves of the Plan (0) (○), Plan (1) (•), Plan (2) (◆) and Plan (3) (■) showing successive levels of dose correction for the PTV and an avoidance structure equal to the phantom minus the PTV volume. Note the increasing slope of the PTV DVH on each successive recursion.

**Figure 3:**
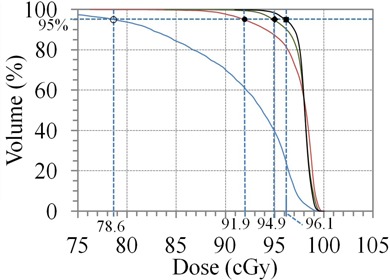
Same data as in Fig. 1, except dose is renormalized to the maximum dose in the PTV. The difference in dose at 95% volume diminishes between successive recursions.

**Figure 4:**
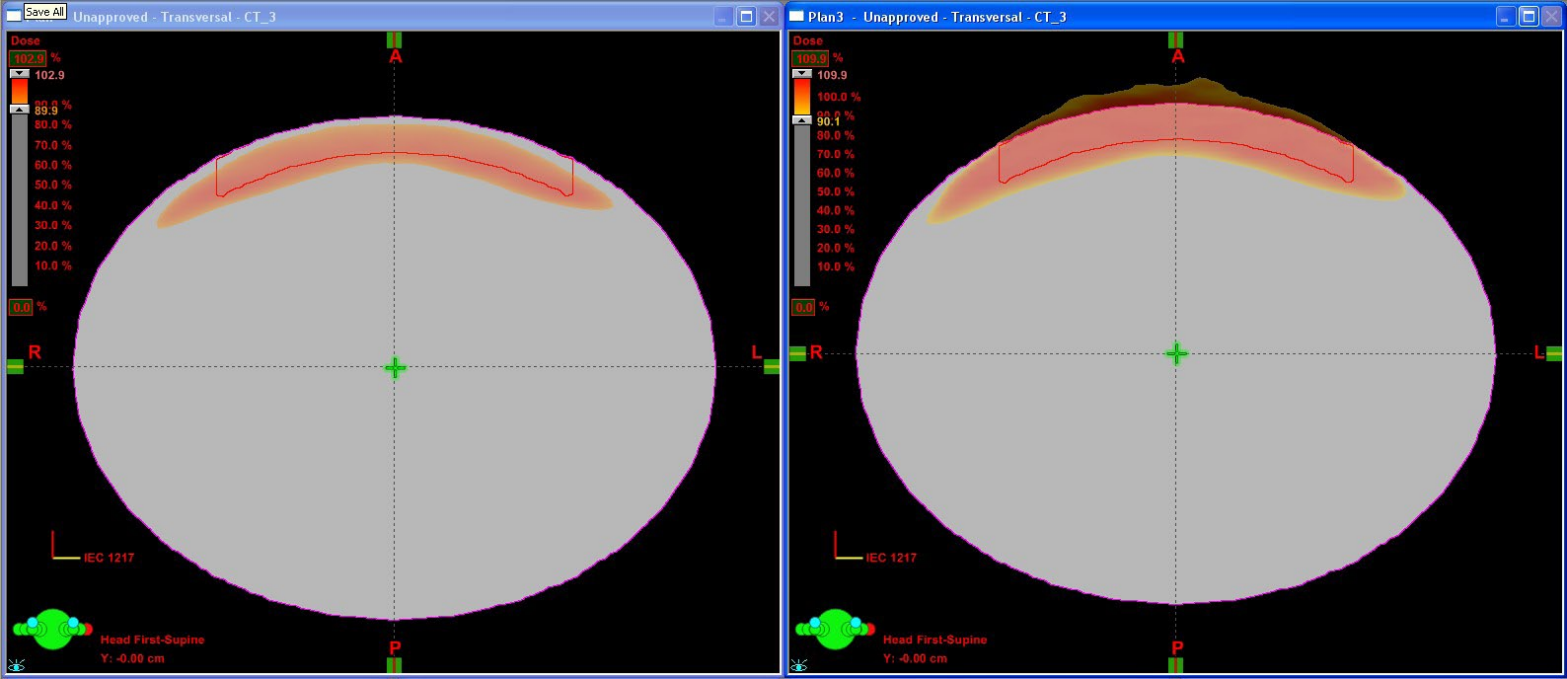
Color wash of Plan (0) with 100 cGy PTV optimizer dose and Plan (3) with PTV optimizer dose 108 cGy. The lower limit is set to 90% for both plans while the upper limit is dependent on plan optimization. It is 102.9 for Plan (0) and 109.9 for Plan (3), reflecting the additional dose added in optimization. Plan (0) has a cold spot on the surface which is typical of OCEs of the Eclipse DVO. Plan (3) has compensated for most of the error in the optimization by the additional dose.

The elliptical water phantom generated in Eclipse contains a superficial PTV with the seven field plan used in this study, as shown in Fig. 1. The DVH curves for the unnormalized Plan (0) and three successive sum plans are shown in Fig. 2. The increase in PTV dose on each iteration is summarized in Table 2. The maximum PTV dose is a sum of the DVO optimizer PTV dose from the previous recursion and the dose correction. For illustrative purposes, the DVH curves may be rescaled to the Max PTV dose, as shown in Fig. 3. The change in the PTV curve between successive recursions becomes sequentially less; the change in dose for the 95% volume point is 1.3% on recursion 3. Fig. 4 shows a color wash of unnormalized Plan (0) and Plan (3) with the lower bound set to 90% of the Rx dose. The dose correction plans add additional dose on the surface of the PTV, which increases the maximum dose from 102.9 for Plan (0) to 109.9 for Plan (3). When the plans are normalized to 100% dose at 95% volume, Plan (0) will have a maximum dose of 127.3 cGy vs. 104.1 cGy for Plan (3). One of the benefits of correcting for optimizer errors is a reduction in maximum dose. The other is that it improves the ability of the optimizer to achieve target dose when more than one PTV exists with differing dose requirements. The unnormalized plans have a cold spot on the surface which extends about 0.2–0.3 cm into the surface near the edge of the PTV. This effect is typical of OCEs generated within the Eclipse DVO.

**Table 2:** Optimizer PTV dose and the maximum PTV dose in the sum plan for three recursions.

*Recursion #*	*Optimizer PTV Dose*	*Max PTV Dose Plan(i)*
0	100	102.93
1	104	104.72
2	106	107.23
3	108	109.87

#### B.1 Clinical case 1: scalp vertex

**Figure 5:**
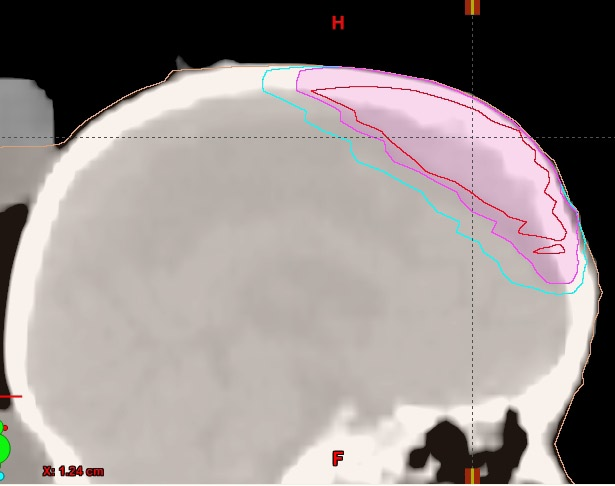
Scalp vertex clinical case. The inner volume is the GTV, followed by the CTV and PTV. The CTV extends to the surface.

**Figure 6:**
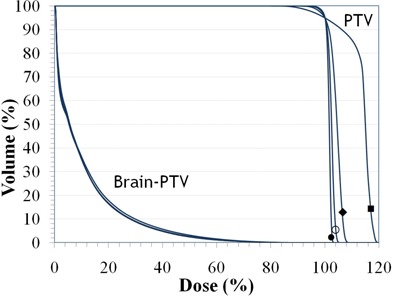
Results for the Scalp Vertex clinical case. DVH curves of the normalized plans Plan (0) (■), Plan (1) (◆), Plan (2) (◆) and Plan (3) (•) showing the improved dose uniformity to the PTV volume while the avoidance structure (Brain‐PTV) remains unaffected.

Fig. 5 illustrates the GTV (inner structure), CTV (medial structure), and PTV (external structure) of a pediatric neuroblastoma with the CTV extended to the surface. Pediatric cases like this are not uncommon. Many of them involve surgical resection. The results of the recursive procedure, shown in Fig. 6, improve the delivery of dose to the surface and reduce the maximum dose from 120 cGy to less than 105 cGy per fraction. The dose to the brain remains unaffected by the additional modulation required to deliver dose to the surface.

#### B.2 Clinical case 2: stereotactic body radiotherapy of a lung tumor

**Figure 7:**
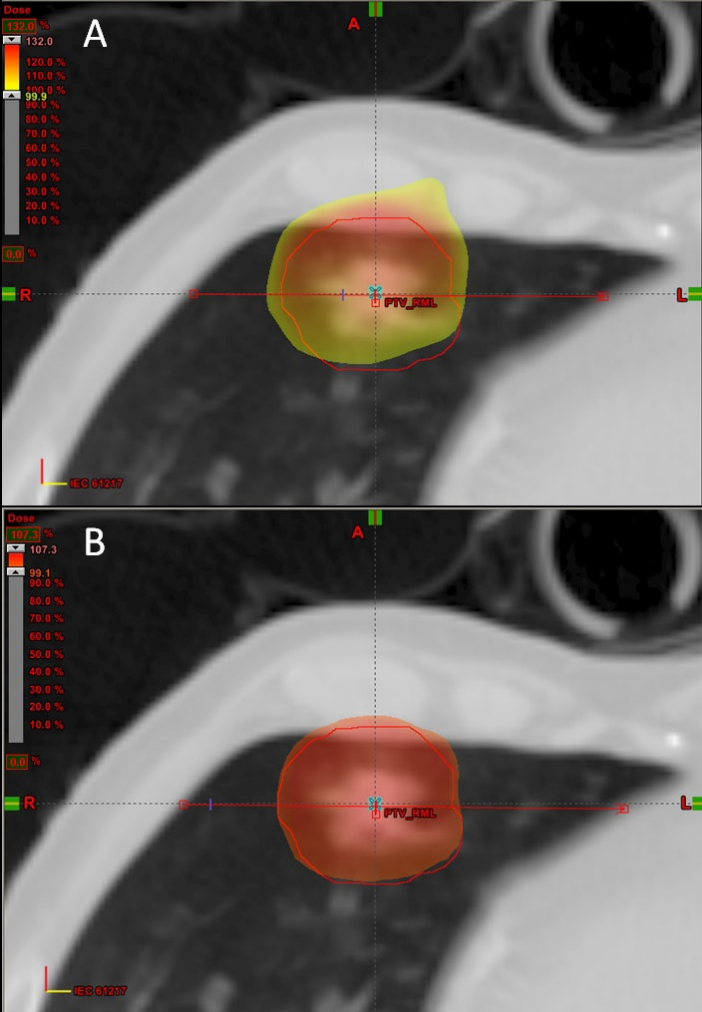
Stereotactic body radiotherapy clinical case. The tumor volume is near the chest wall in the right medial lung. Top figure: original uncorrected plan; bottom figure: corrected plan (Plan (2)) showing improved dose conformity.

**Figure 8:**
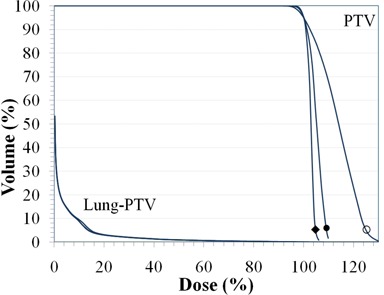
DVH curves of the normalized plans Plan (0) (■), Plan (1) (◆), and Plan (2) (○) showing the improved dose uniformity to the PTV volume while the avoidance structure (Lung‐PTV) remains unaffected.

The magnitude of correction for lateral scatter in the optimizer depends on the location of the tumor volume in the lung. Near the chest wall, the correction is less compared to a centrally located tumor. Figure 7 illustrates the dose distribution for Plan 0 and Plan 2 normalized to the 95% volume point for a typical case of a tumor volume near a chest wall. The lower bound on the color wash is set to 100% in both cases. Note the improved conformity of dose which reduces the dose to the adjacent chest wall, important in reducing complications. Figure 8 illustrates the change in DVH for the PTV as well as the lung volume. The maximum dose in the PTV is reduced from 130 cGy to 105 cGy while the lung volume remains unaffected.

The cases where this procedure is applied need to be carefully considered due to some additional workload. It is not beneficial to correct prostate plans, for example. Head and neck cases may benefit depending on how near to the surface the physician segments the PTV volume. Superficial lesions and lung cases almost always benefit from this procedure. The time required to implement this procedure is approximately twice that of a conventional optimization, as each successive recursion uses the same optimization parameters while requiring fewer iterations. There is some additional time in exporting and converting files and importing them back into Eclipse, but this time is small compared to the time required for the initial optimization.

## IV. CONCLUSIONS

This iterative and recursion procedure corrects for errors originating in the Eclipse DVO and in the modeling of the intensity modulator to provide an optimized plan that is consistent with the accuracy of the Eclipse AAA final dose calculation algorithm. The procedure may be implemented with the base plan feature of Eclipse and a simple spreadsheet program provided in this technical note to add the optimized fluence maps. AAA has been shown to be a very accurate algorithm for IMRT, with the caveat that the PTV should be deeper than the dose buildup region. Most treatment planning systems utilize fast coding in the optimizer and perform an accurate final dose calculation. It is therefore desirable for this procedure to be implemented within the optimizer of these treatment planning systems to make their optimization process consistent with final dose calculation. This procedure may also hold value respecting other treatment planning systems, with the benefit dependent on how well the optimizer code is written. The AAA may still have some error when compared to Monte Carlo. Ideally, Monte Carlo could provide the final dose calculation while using the proposed corrective procedure to maintain the speed of the optimizer.

## Supporting information

Supplementary Material Files

Supplementary Material Files
